# Cooperative transport by flocking phototactic micromotors†

**DOI:** 10.1039/d1na00641j

**Published:** 2021-09-02

**Authors:** Jianhua Zhang, Fangzhi Mou, Zhen Wu, Jiaqi Song, Joshua E. Kauffman, Ayusman Sen, Jianguo Guan

**Affiliations:** State Key Laboratory of Advanced Technology for Materials Synthesis and Processing, International School of Materials Science and Engineering, Wuhan University of Technology 122 Luoshi Road Wuhan 430070 China moufz@whut.edu.cn; Department of Chemistry, The Pennsylvania State University University Park PA 16802 USA

## Abstract

Cargo delivery by micro/nanomotors provides enormous opportunities for micromanipulation, environmental cleaning, drug delivery, *etc.* However, due to the limited driving force, it is usually difficult for single micro/nanomotors to transport cargoes much larger or heavier than themselves. Here, we demonstrate that flocking phototactic TiO_2_ micromotors can cooperatively transport multiple and different types of large cargoes based on light-responsive diffusiophoresis. Utilizing spontaneous diffusiophoretic attraction, flocking TiO_2_ micromotors can load large cargoes. Under UV light navigation, flocking TiO_2_ micromotors cooperatively carry and transport cargoes *via* collective diffusiophoretic repulsion in open space or complex microenvironments. After reaching the destination, the carried cargoes can also be unloaded from the flock and be deployed at a predetermined destination by disassembling or reversing the flock. This study may pave the way for developing intelligent swarming micro/nanorobots for cooperative targeting micromanipulation and advancing their applications in drug delivery and microengineering.

## Abstract 2

Flocking TiO_2_ micromotors are able to cooperatively manipulate multiple and different types of large cargoes under the photochemical navigation of UV light.

## Graphical abstract

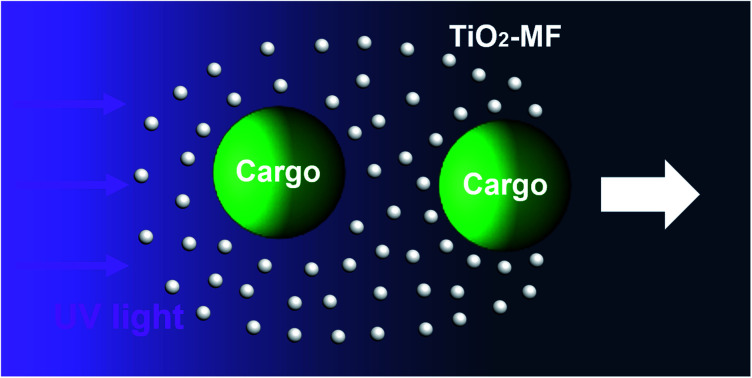

## Introduction

Controllable capture and transport cargoes such as drug molecules, proteins, capsules, and cells in microenvironments is a pivotal nanotechnology that is extensively studied.^1–6^ Synthetic micro/nanomotors can extract various energies from the surrounding environment, and convert them into mechanical output allowing for autonomous motions.^7–15^ Thus, micro/nanomotors are endowed with the undeniable capability to serve as micro/nanocarriers to realize micromanipulation by establishing physical/chemical interactions between the motors and cargoes.^16–18^ Up to now, cargo delivery *via* single micro/nanomotors has been investigated extensively by introducing diffusiophoretic, electrostatic, and magnetic motor–cargo interactions through chemical-powering^19–21^ and external-field-driven strategies.^22–27^ However, the magnitude of the generated driving forces of single motors would be seriously hindered by their dimensions, thus larger or heavier cargoes are difficult to transport. Inspired by the ant army carrying food cooperatively, the driving force would be much enhanced by grouping the motors.^28–32^ Therefore, growing endeavors have been made to construct intelligent micro/nanorobot swarms to cooperatively transport large cargoes.^33^ The key to achieving cooperative transport is to align the forces of active individuals in swarms in the same direction. For example, reconfigurable magnetic motor swarms can regulate their forces effectively in elaborated magnetic fields, thereby manipulating passive cargoes by utilizing hydrodynamic flow.^34,35^ Chemically/photochemically powered micro/nanomotors can respond to signaling chemicals secreted by their neighbors and behave as motor groups utilizing local diffusiophoretic interactions.^36–42^ Nevertheless, aligning the forces of these individuals to effectively manipulate multiple and different types of large cargoes is still an insurmountable challenge.

In this study, we demonstrate the cooperative transport of multiple and different types of large cargoes by TiO_2_ micromotor flocks (TiO_2_-MFs) under UV navigation. Specifically, isotropic TiO_2_ micromotors with rich hydroxyl groups can autonomously form clusters based on electrolyte diffusiophoretic attraction, which is also capable of capturing heterogeneous passive large cargoes. Meanwhile, the non-electrolyte diffusiophoretic repulsive force resulting from the photodegrading H_2_O_2_ by the flocking TiO_2_ motors can be aligned toward a determined direction against the UV light. Thus, TiO_2_-MFs, even loaded with multiple cargoes, can exhibit negative phototaxis with intense collective expansion. Even though TiO_2_-MFs slow down gradually with increasing loads of cargoes, they can transport up to nine large cargoes. In addition, the carried cargoes can also be unloaded from the flock and be deployed at a designated destination by disassembling the flock into highly dispersed micromotors under intense long-time UV irradiation or by reversing the flock away from cargoes. TiO_2_-MFs show broad applicability and excellent feasibility for loading and ferrying different types of large cargoes (SiO_2_ microspheres, amino-polystyrene microspheres, carboxyl-polystyrene microspheres and perfluorooctane droplets) benefitting from the universal light-switchable motor–cargo interactions (diffusiophoretic attraction and repulsion). The cooperative transport of large cargoes by TiO_2_-MFs in microchannels has also been investigated, manifesting the possibility of realizing cargo transport in microenvironments with complex landscapes. The as-proposed cooperative transport strategy may open a window for the development of general transport systems based on swarming micro/nanorobots to deliver different functional micro/nanoobjects, such as drug capsules, micro-droplets, micromachinery parts for drug delivery, micromanipulation, and microengineering.

## Experimental section

### Materials

Silica, carboxyl-polystyrene, and amino-polystyrene microparticles, as large cargoes, were purchased from Sigma-Aldrich (Shanghai, China), and perfluorooctane was purchased from Aladdin Chemistry Co., Ltd. (Shanghai, China). Tetrabutyl titanate (TBT) was purchased from Sinopharm Chemical Reagent (Shanghai, China).

### Synthesis of TiO_2_ micromotors

The constituent isotropic TiO_2_ micromotors used in this work were prepared following previous reports.^26,43^ Typically, 25 mL ethanol was mixed with 100 µL sodium chloride aqueous solution (0.1 M) and 425 µL TBT at ambient temperature. After magnetic stirring for 18 min, the solution was allowed to stand for 24 h. The resulting precipitate was separated, washed three times with alcohol and deionized water, and then dried at 60 °C for 12 h to obtain amorphous TiO_2_ microspheres. Finally, anatase TiO_2_ micromotors were obtained *via* calcinating amorphous TiO_2_ at 300 °C for 2 h.

### Cooperative transport

A 50 µL suspension of the TiO_2_ micromotors (1 mg mL^−1^) was dropped onto a glass slide (Citotest 1A5107), followed by adding 50 µL H_2_O_2_ fuel solution (0.75 wt%) and 50 µL cargo microparticle suspension (0.03 mg mL^−1^). The dispersed TiO_2_ micromotors usually spontaneously gather with the cargo microparticles and form into clusters within five minutes. Four UV-LED light sources (SZ Lamplic Technology, China) with a wavelength of 365 nm and adjustable intensity (0–500 mW cm^−2^) were set above the glass substrate with an incident angle of 45°, as demonstrated in the experimental setup in Fig. S2.† The on/off and incident direction of the UV light were adjusted to investigate the phototaxis of TiO_2_-MFs and their capability for cooperatively transporting large cargoes. The experimental results were observed and recorded at room temperature using an inverted optical microscope (Leica DMI 3000 M, Germany). Videos were analyzed by using ImageJ and Video Spot Tracker V08.01 software. The velocity of the flocks was determined by calculating the displacement of centroid of flocks per second under light irradiation. Over four TiO_2_-MFs were analyzed to obtain the statistic result. The error bar given in the plot is the standard error of the mean.

### Numerical simulation

Numerical simulations were performed using the diffusion module of COMSOL Multiphysics software. The simulation model was built up by immersing a 10 µm SiO_2_ microsphere surrounded by 65 TiO_2_ micromotors (1.2 µm in diameter (*d*)) in the bottom of a 0.01 mm^2^ square space which was filled with H_2_O_2_ aqueous solution (0.25 wt%). The flux (*J*_O_2__) of oxygen molecules from the UV illuminated surface of the TiO_2_ micromotors was set as 4.13 × 10^−4^ mol m^−2^ s^−1^ according to the previous report.^43^ The diffusion coefficient of O_2_ molecules in water was set to be 1.97 × 10^−9^ m^2^ s^−1^. The distribution of O_2_ concentration was simulated at the steady state, and the average interparticle distance (*L*) was derived from the experimental results of the collective expansion of TiO_2_-MFs under UV irradiation.

## Results and discussion

**Fig. 1:**
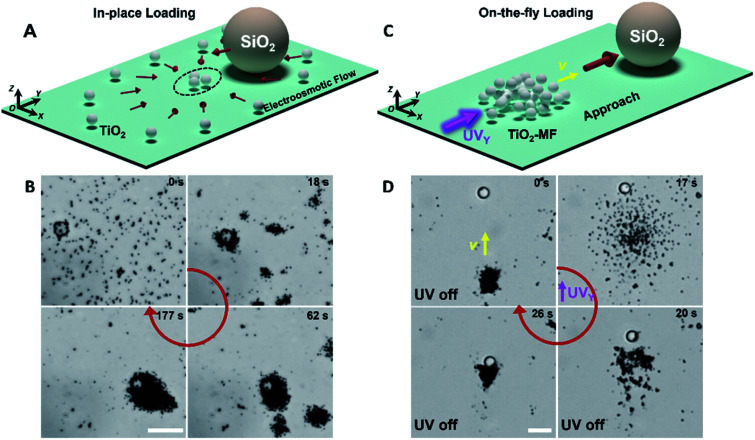
Two modes of large-cargo loading by TiO_2_-MFs. (A and B) The schematic diagram (A) and optical microscope snapshots (B) depicting the in-place large-cargo loading utilizing spontaneous swarming of TiO_2_ micromotors (*d*: 1.2 µm) together with a large SiO_2_ microsphere (*d*: 10 µm). (C and D) The schematic diagram (C) and optical microscope snapshots (D) depicting the on-the-fly large-cargo loading by steering a TiO_2_-MF to approach a large SiO_2_ microsphere (10 µm). The yellow arrow indicates the motion direction of the flock. H_2_O_2_ concentration: 0.25 wt%. Scale bars in (B) and (D): 20 µm.

The constituent isotropic TiO_2_ micromotors (1.2 µm in *d*) of TiO_2_-MFs were fabricated referring to the previous work.^26^ According to the reported mechanism, the TiO_2_ micromotors with rich hydroxyl groups secrete H^+^ and OH^−^ ions in aqueous media, and thus can spontaneously gather into clusters due to the diffusiophoretic motor–motor attraction without external energy input.^43^ It is noted that this diffusiophoretic attraction can also compel adjacent passive particles to assemble with the TiO_2_ micromotors. Fig. 1A shows the schematic diagram depicting the spontaneous in-place loading of a large SiO_2_ microsphere (10 µm) during the gathering of the dispersed TiO_2_ micromotors. As shown in the corresponding experimental microscope snapshots in Fig. 1B, the TiO_2_ micromotors aggregate gradually to form small clusters, and then these small clusters further merge into large clusters along with time progression (ESI Video 1†). Remarkably, multiple large SiO_2_ microspheres could be loaded by the swarming TiO_2_ micromotors (Fig. S1†).

Like other photochemical TiO_2_-based micromotors, the gathered TiO_2_ micromotors can be powered by photocatalytically decomposing surrounding H_2_O_2_ fuels.^44–50^ Under sidewise UV irradiation, the gathered TiO_2_ micromotors exhibit negative phototaxis as a flock (TiO_2_-MF) in the medium with the H_2_O_2_ fuel,^43,44^ and thus can also be driven to approach and load a large cargo on the fly. The on-the-fly cargo loading is illustrated in Fig. 1C and D (taken from ESI Video 2†) shows the corresponding experimental results. Under the navigation of sidewise UV light (UV_*Y*_ in Fig. 1D), the TiO_2_-MF moved toward a SiO_2_ microsphere utilizing its dilatational negative phototaxis based on the dominant nonelectrolyte diffusiophoresis (0–17 s in Fig. 1D), and finally loaded it into the flock *via* the electrolyte diffusiophoretic attraction after removing the UV_*Y*_ (20–26 s in Fig. 1D).

**Fig. 2:**
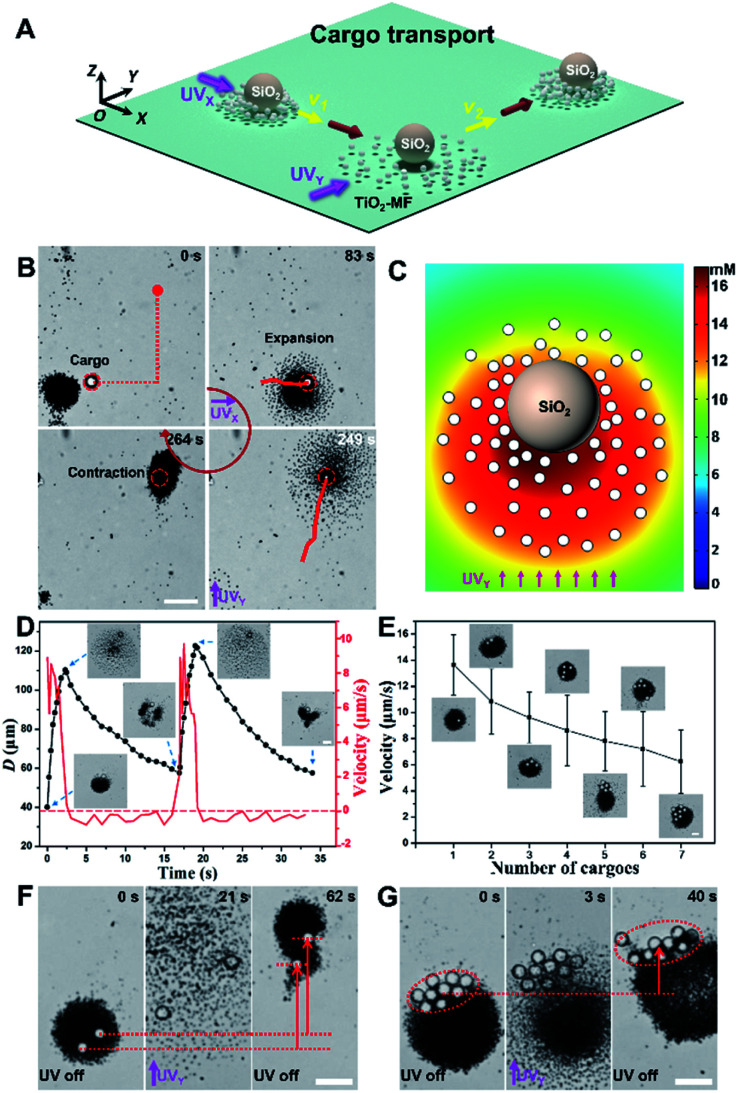
Cooperative large-cargo transport by flocking phototactic TiO_2_ micromotors. (A) Schematic diagram of light-controlled directional cooperative transport of large cargoes by operating the TiO_2_-MF. (B) A SiO_2_ microparticle (*d*: 10 µm) was delivered by a TiO_2_-MF along a pre-designed path under the navigation of pulsed UV irradiation. Images are taken from ESI Video 3.† Scale bar: 10 µm. (C) Simulated O_2_ concentration around a passive SiO_2_ microsphere surrounded by 65 TiO_2_ micromotors in H_2_O_2_ aqueous solution (0.25 wt%) under UV irradiation (500 mW cm^−2^). The interparticle distance was derived from the experimental results on transport. (D) The size (*D*) variation of a typical flock and the velocity variation of the loaded SiO_2_ microsphere *versus* time under the pulsed UV irradiation. The insets depict the states of the flock and the cargo at various moments. Scale bar: 10 µm. (E) Average motion velocity of typical TiO_2_-MFs (with similar *D*) carrying different numbers of cargoes (SiO_2_ microspheres with a *d* of 10 µm) under UV light illumination. The insets illustrate the microscopic images of typical flocks loading with different numbers of large cargoes. The error bar of each data point given in the plot is the standard error of the mean of over four different TiO_2_-MFs. Scale bar: 10 µm. (F and G) The optical microscope snapshots of cooperative transport of two (F) and nine (G) large SiO_2_ microspheres by TiO_2_-MFs with UV manipulation. Images are taken from ESI Video 4.† Scale bar: 20 µm. UV light intensity: 500 mW cm^−2^. H_2_O_2_ concentration: 0.25 wt%.

After being loaded by a TiO_2_-MF, the large cargo can then be transported because it also feels the electrolyte/nonelectrolyte gradients generated by active TiO_2_ micromotors, suggesting that it will move and gather with the flocking TiO_2_ micromotors (in resemblance to a failed constituent in the flock) when the UV light is on and off, respectively. In other words, the active transport of passive cargoes can be achieved by operating TiO_2_-MFs *via* UV light. Fig. 2A and B depict the schematic diagram and experimental snapshots of the cooperative transport of a large SiO_2_ microsphere (*d*: 10 µm) by a TiO_2_-MF along the pre-designed path (red dashed lines in Fig. 2B) to a predetermined destination (ESI Video 3†). In detail, when turning on the UV_*X*_ (UV light is toward the +*X* axis direction), the large SiO_2_ microsphere was pushed expansively *via* nonelectrolyte diffusiophoretic repulsion of the TiO_2_-MF. Once removing the light, the cargo was dragged back to form a resting flock with TiO_2_ micromotors again at a new central point and showed a net phototactic displacement. When the UV_*Y*_ (UV light is toward the +*Y* axis direction) was then turned on, the flock exhibited an immediate turning toward the +*Y* direction. The O_2_ distribution around a large SiO_2_ microsphere surrounded by 65 TiO_2_ micromotors in the transport process was simulated by using the diffusion module of COMSOL Multiphysics software. As illustrated in Fig. 2C, upon UV_*Y*_ irradiation, a distinct O_2_ concentration gradient forms across the large cargo which can lead to the non-electrolyte diffusiophoresis to propel the cargo. Fig. 2D shows the diameter (*D*) variation of a typical TiO_2_-MF and the velocity variation of the loaded cargo (the large SiO_2_ microsphere) *versus* time under two pulsed UV_*Y*_ irradiation, where the instantaneous velocity vector subtly reflects the corresponding pushing and dragging processes discussed above. The cargo moved forward (positive velocity of red curve in Fig. 2D) with the expanding TiO_2_-MF (insets and black curve in Fig. 2D) under UV illumination, and then slightly moved backward (negative velocity of the red curve in Fig. 2D) when the TiO_2_-MF contracted back to a tight resting flock in the absence of UV light (insets and black curve in Fig. 2D). The agreement of these two curves in time proves the synchronized motions of the TiO_2_-MF and the cargo.

More interestingly, the TiO_2_-MF can also transport multiple large cargoes due to its strong driving force (Fig. 2E). Insets in Fig. 2E are the microscope snapshots showing TiO_2_-MFs with similar *D* (*ca.* 50 µm) loaded with different numbers of large cargoes (SiO_2_ microspheres). Under the same light and fuel conditions, these TiO_2_-MFs moved slower when carrying more SiO_2_ microspheres. Specifically, the average motion velocity of TiO_2_-MFs decreased from 13.7 to 6.2 µm s^−1^ when the number of the carried SiO_2_ microspheres increased from one to seven. The decreasing velocity can be rationalized by the greater burden if more passive cargoes were carried while the powering parts (TiO_2_ micromotors) were unchanged. The slight difference in the size of TiO_2_-MFs was believed to have a negligible influence on the velocity of these flocks as they had a similar velocity (Fig. S3†). The light-controlled cooperative transport of multiple large cargoes by TiO_2_-MFs is further visually illustrated in Fig. 2F and G (taken from ESI Video 4†), and the displacements in the +*Y* direction were clearly observed whether the transported cargo number was two or nine (red arrows in Fig. 2E and F).

**Fig. 3:**
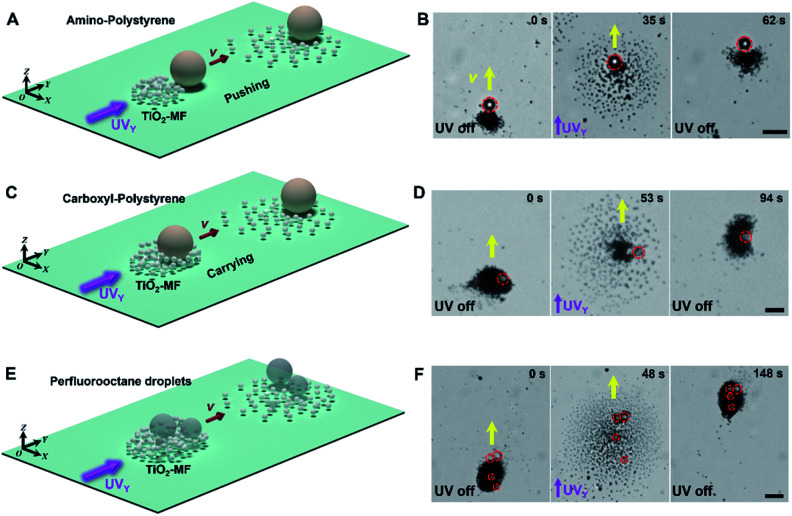
Cooperative transport of different types of large cargoes by flocking phototactic TiO_2_ micromotors. The schematic diagrams and optical microscope snapshots depicting the cooperative transport of an amino-polystyrene microsphere ((A) and (B), *d*: 10 µm), a carboxyl-polystyrene microsphere ((C) and (D), *d*: 10 µm) and perfluorooctane droplets ((E) and (F), the average *d* is 5 µm) by TiO_2_-MFs. The red dashed circles show the positions of the corresponding cargo particles. Yellow arrows indicate the motion direction of the flocks. Images are taken from ESI Video 5.† Scale bars: 10 µm. UV light intensity: 500 mW cm^−2^. H_2_O_2_ concentration: 0.25 wt%.

Besides, TiO_2_-MFs can cooperatively transport different types of heavy cargoes, such as amino- and carboxyl-polystyrene microspheres, and perfluorooctane droplets (Fig. 3). Similar to the spontaneous loading process of the large SiO_2_ microsphere (Fig. 1A and B), three different types of large cargoes, including an amino-polystyrene microsphere (*d*: 10 µm), a carboxyl-polystyrene microsphere (*d*: 10 µm) and perfluorooctane droplets (average *d*: 5 µm) can be loaded into the TiO_2_-MF because of the electrolyte diffusiophoretic attraction, and then exhibited synchronized negative phototaxis with TiO_2_-MFs under pulsed UV_*Y*_ light (ESI Video 5†). Fig. 3A, C and E depict the schematic diagrams of transporting corresponding cargoes. The red dashed circles in Fig. 3B, D and F indicate the real-time positions of the cargo particles. These results prove the good adaptability of TiO_2_-MFs, which guarantees the ability to be used in different application scenarios.

**Fig. 4:**
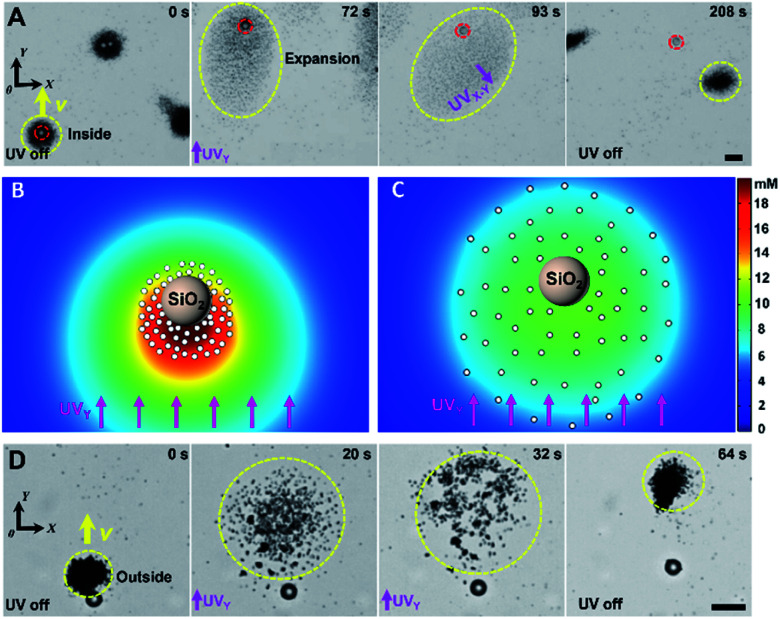
Light-controlled large-cargo unloading from TiO_2_-MFs. (A) Time-lapse optical microscopy images illustrating the unloading of a SiO_2_ microsphere that was inside of a TiO_2_-MF by disassembling the flock, taken from ESI Video 6.† (B and C) Simulated O_2_ concentration around a passive SiO_2_ microsphere surrounded by 65 TiO_2_ micromotors in H_2_O_2_ aqueous solution (0.25 wt%) under UV irradiation (500 mW cm^−2^) with different *L* of 2.5 (B) and 6 µm (C). The different *L* is because of the collective expansion based on nonelectrolyte diffusiophoresis upon UV illumination. (D) Time-lapse optical microscopy images illustrating the unloading of an amino-polystyrene microsphere that was outside of a TiO_2_-MF by reversing the flock away from the microsphere, taken from ESI Video 7.† Yellow and red dashed circles represent objects of TiO_2_-MFs and cargoes, respectively. Yellow arrows indicate the motion direction of the flocks. Scale bars: 10 µm. UV light intensity: 500 mW cm^−2^, H_2_O_2_ concentration: 0.25 wt%.

Once arriving at a predesigned destination, large cargoes can be unloaded from TiO_2_-MFs by modulating UV light. Two modes of cargo unloading can be realized depending on the different relative positions of the cargoes and TiO_2_-MFs, including disassembling and reversing the flock, as shown in the experimental results in Fig. 4A and B. For a large cargo that was carried inside of a TiO_2_-MF, it can be unloaded by disassembling the flock. Specifically, when a SiO_2_ microsphere (as the large cargo, 10 µm) was carried to the destination under the navigation of the pulsed UV_*Y*_ irradiation (0–72 s in Fig. 4A), an extra UV light (UV_*X*_) was turned on. Under the superimposed continuous UV_*X*_ and UV_*Y*_ irradiation (or the continuous UV_*X*–*Y*_ irradiation in a direction with an angle of 45° between the +*X* and −*Y* axis), the TiO_2_-MF was disassembled into scattered micromotors. With the prolonging irradiation time, the scattered TiO_2_ micromotors moved away from the large cargo (72–93 s in Fig. 4A). Thus, a distinct displacement difference was induced between the TiO_2_-MF and cargo (93 s in Fig. 4A), and the large cargo was then unloaded because the electrolyte diffusiophoretic attraction became too weak due to the large flock-cargo distance after the UV_*X*–*Y*_ irradiation was off (208 s in Fig. 4A). The yellow and red dashed circles in Fig. 4A mark the real-time positions of the TiO_2_-MF and the cargo, respectively, which also record the corresponding unloading process. To decipher the cargo unloading by disassembling the TiO_2_-MF, we simulated the distribution of the photogenerated O_2_ molecules across the large cargo surrounded by a cluster of TiO_2_ micromotors (1.2 µm) with different average motor–motor distances (*L*). With increasing *L* from 2.5 (Fig. 4B) to 6 µm (Fig. 4C), corresponding to the different scattered states of the TiO_2_ micromotors under normal UV_*Y*_ irradiation (72 s in Fig. 4A) and under continuous UV_*X*–*Y*_ irradiation (93 s in Fig. 4A), the O_2_ concentration gradient across the SiO_2_ microsphere along the +*Y* direction decreased sharply, weakening its nonelectrolyte diffusiophoresis (or the subjected diffusiophoretic pushing force from the micromotors). As a result, the SiO_2_ cargo is left behind by the scattered TiO_2_ micromotors and finally unloaded from the TiO_2_-MF. On the other hand, in the scenario that the large cargo is outside of the TiO_2_-MF, it would be easier for the cargo to be unloaded. As shown in Fig. 4D, with the UV irradiation pointing in the +*Y* direction, the TiO_2_-MF would move backward away from the carried cargo (an amino-polystyrene microsphere, 10 µm), and then the cargo was unloaded from the flock successfully.

**Fig. 5:**
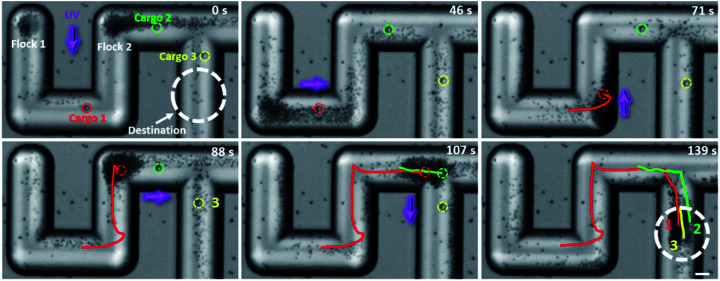
Time-lapse optical microscopy images illustrating the cooperative transport of three large cargoes by TiO_2_-MFs under the navigation of UV light in a microchannel. The cargoes (SiO_2_ microspheres, 10 µm) are marked in different colors. The white dashed circles represent the target area. The optical microscope snapshots are taken from ESI Video 8.† Scale bar: 20 µm. UV light intensity: 500 mW cm^−2^. H_2_O_2_ concentration: 0.25 wt%.

The transport capability of TiO_2_-MFs in a confined space was also tested here, as demonstrated in Fig. 5 and ESI Video 8.† When the TiO_2_ micromotors and three large cargoes (SiO_2_ microspheres, 10 µm) were transferred into a glass microchannel in a rotated S-shape, they formed two flocks at different corners, and the three cargoes (circled by red, green, and yellow circles, respectively) were located at different positions. In the presence of UV_*Y*_ (−*Y* direction), the two TiO_2_-MFs initiated negative phototaxis toward the same wall and merged into a large flock (0–46 s). In the meantime, cargo 1 was loaded by the TiO_2_-MF. After turning the UV light to +*X* direction, the merged TiO_2_-MF coercing cargo 1 moved toward the right corner. By a similar approach, the TiO_2_-MF was guided to pass through the “S” microchannel and deliver the cargoes to the pre-designed destination within 139 s. The red, green and yellow lines in Fig. 5 depict the trajectories of cargoes 1, 2, and 3, respectively. The above results reveal the feasibility of cooperative transport in complex landscapes by the flocking TiO_2_ micromotors.

## Conclusions

This work has demonstrated that phototactic TiO_2_-MFs can cooperatively manipulate multiple and different types of large cargoes under UV navigation. The swarming and collective phototaxis of TiO_2_ micromotors are produced from the spontaneous electrolyte diffusiophoretic attraction and photo-induced nonelectrolyte diffusiophoretic repulsion, which can also be tuned to load, transport, and release large cargoes when controlling the on/off, illumination time and incident direction of UV light. Thus, under UV navigation, multiple and different types of large cargoes can be cooperatively transported along a predesigned path and be deployed at a predetermined destination in an open space or complex microenvironments. This strategy of cooperative transport may advance the applications of swarming micro/nanomotors in drug delivery, micromanipulation and microengineering.

## Conflicts of interest

There are no conflicts to declare.

## Supplementary Material

NA-003-D1NA00641J-s001

NA-003-D1NA00641J-s002

NA-003-D1NA00641J-s003

NA-003-D1NA00641J-s004

NA-003-D1NA00641J-s005

NA-003-D1NA00641J-s006

NA-003-D1NA00641J-s007

NA-003-D1NA00641J-s008

NA-003-D1NA00641J-s009
