# Supplementary material for: Cooperative transport by flocking phototactic micromotors
Source: Nanoscale Adv. 2021 Sep 2;3(21):6157–63. doi: 10.1039/d1na00641j (PMC9419550; doi:10.1039/d1na00641j)
Supplement: NA-003-D1NA00641J-s001 [file NA-003-D1NA00641J-s001.pdf]

Electronic Supplementary Material (ESI) for Nanoscale Advances.
This journal is © The Royal Society of Chemistry 2021

Electronic Supplementary Material (ESI) for Nanoscale.

This journal is © The Royal Society of Chemistry 2019

Supporting Information

Cooperative transport by flocking phototactic micromotors

Jianhua Zhang,[ab] Fangzhi Mou,*[a] Zhen Wu,[a] Jiaqi Song,[b] Joshua E. Kauffman,[b] Ayusman Sen[b] and Jianguo Guan[a]

[a]State Key Laboratory of Advanced Technology for Materials Synthesis and Processing, International School of Materials Science and Engineering, Wuhan University of Technology, 122 Luoshi Road, Wuhan 430070, China. E-mail: moufz@whut.edu.cn

[b]Department of Chemistry, The Pennsylvania State University, University Park, PA 16802, USA

Supporting Figures

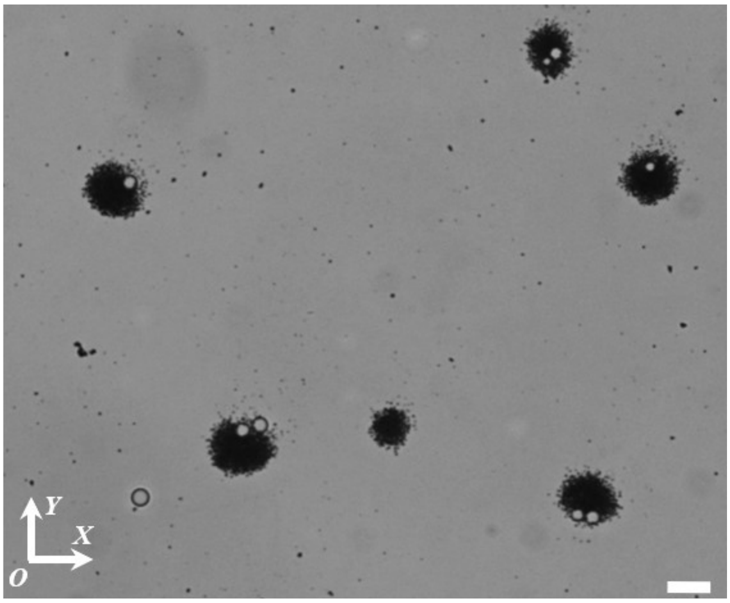

Figure S1. The microscope image of the TiO_2-MFs loaded with cargoes (SiO_2 microspheres, average diameter: 10 μm). Scale bar: 20 μm.

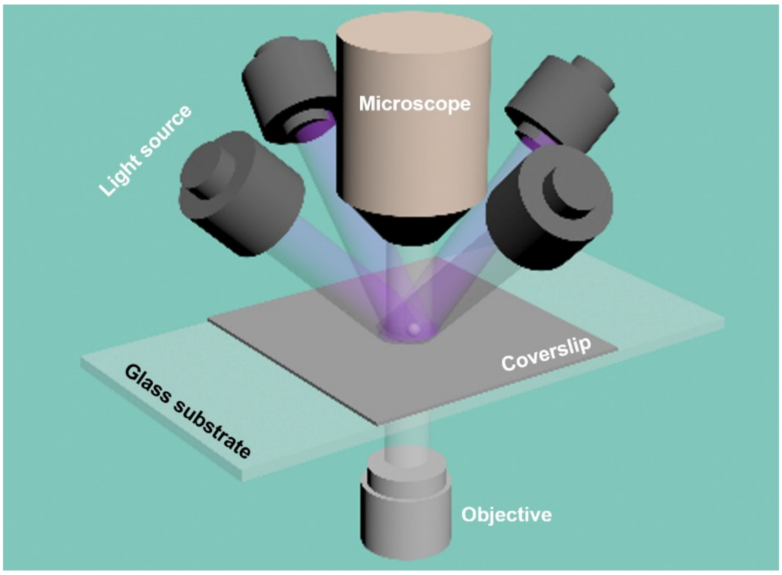

Figure S2. Schematic diagram of the experimental setup.

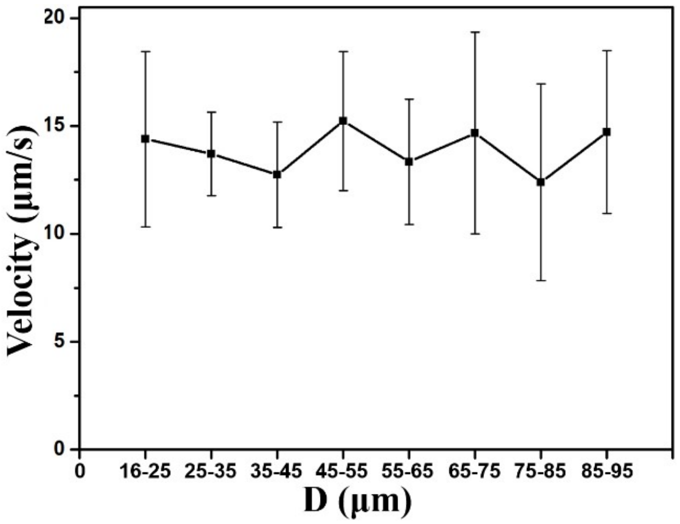

Figure S3. The average motion velocity of TiO_2-MFs *versus* various diameters (*D*) under the constant condition of UV intensity (500 mW cm^{-2}) and H_2O_2 concentration (0.25 wt.%).

Supporting Videos

Video 1: The autonomous swarming of a large SiO_2 microsphere and concentrated TiO_2 micromotors near the glass substrate in H_2O_2 aqueous solution (0.25 wt.%).

Video 2: Light-controlled, on-the-fly loading of a large SiO_2 microsphere (10 μm) by optically controlling a TiO_2-MF, with light intensity of 500 mW cm^{-2} and in 0.25 wt% H_2O_2.

Video 3: Light-controlled cooperative transport of a large SiO_2 microsphere by optically controlling a TiO_2-MF, with light intensity of 500 mW cm^{-2} and in 0.25 wt% H_2O_2.

Video 4: Light-controlled cooperative transport of two and nine SiO_2 microspheres by optically controlling the TiO_2-MFs, with light intensity of 500 mW cm^{-2} and in 0.25 wt% H_2O_2..

Video 5: Light-controlled cooperative transport of a large amino-polystyrene microsphere, a large carboxyl-polystyrene microsphere, and perfluorooctane droplets by optically controlling the TiO_2-MF, with light intensity of 500 mW cm^{-2} and in 0.25 wt% H_2O_2.

Video 6: Light-controlled unloading of a large SiO_2 microsphere from the inside of a TiO_2-MF, with light intensity of 500 mW cm^{-2} and in 0.25 wt% H_2O_2.

Video 7: Light-controlled unloading of an amino-polystyrene microsphere from the outside of a TiO_2-MF, with light intensity of 500 mW cm^{-2} and in 0.25 wt% H_2O_2.

Video 8: Light-controlled collective cooperative transport of three large SiO_2 microspheres in a microchannel, with light intensity of 500 mW cm^{-2} and in 0.25 wt% H_2O_2.

Governing equations for numerical simulation.

Upon UV illumination, the prepared TiO_2 micromotors will initiate photocatalytic decomposition of hydrogen peroxide and produce amounts of O_2 molecules. The concentration gradients of O_2 molecules contributing for nonelectrolyte diffusiophoresis were simulated using the diffusions and creeping flow modules of commercial COMSOL Multiphysics package. The distribution of O_2 molecules is governed by the diffusion and convection of O_2 flux (J_{O_2}) (Equation 1). In our model, it is solved with the conservation equation (Equation 2) at steady state.[1,2]

$$J_{O_2} = uc_{O_2} - D_{O_2}\nabla c_{O_2} - \frac{z_{O_2}FD_{O_2}c_{O_2}\nabla_{\varphi}}{RT} \quad (1)$$

$$\nabla \cdot J_{O_2} = 0 = u \cdot \nabla c_{O_2} - D_{O_2}\nabla^2 c_{O_2} - \frac{z_{O_2}FD_{O_2}\nabla\cdot(c_{O_2}\nabla\varphi)}{RT} \quad (2)$$

Of which, u is the fluid velocity, F is the Faraday constant, φ is the electrostatic potential, R is the gas constant, T is the absolute temperature, c_{O_2}, D_{O_2}, and z_{O_2} represent the concentration, diffusion coefficient and charge of O_2 molecules in water, respectively. In addition, the inertial effect is neglected, the fluid is treated as incompressible, and initial values of the flow velocity and the pressure are all zero in the present study.

References

1 J. Zhang, J. Song, F. Mou, J. Guan and A. Sen, *Trends Chem.*, 2021, **3**, 387.

2 H. Xu, M. Medina-Sanchez and O. G. Schmidt, *Angew. Chem. Int. Ed.*, 2020, **59**, 15029.
